# Endogenous Ribonucleases: Therapeutic Targeting of the Transcriptome Through Oligonucleotide-Triggered RNA Inactivation

**DOI:** 10.3390/biom15070965

**Published:** 2025-07-04

**Authors:** Daria A. Chiglintseva, Olga A. Patutina, Marina A. Zenkova

**Affiliations:** Laboratory of Nucleic Acids Biochemistry, Institute of Chemical Biology and Fundamental Medicine SB RAS, 630090 Novosibirsk, Russia; dashachiglintseva@gmail.com (D.A.C.); marzen@niboch.nsc.ru (M.A.Z.)

**Keywords:** RNA-targeted therapy, RNase H1, RNase P, AGO2, antisense oligonucleotide, EGS, siRNA, miRNA, tsRNA, piRNA

## Abstract

The selective regulation of gene expression at the RNA level represents a rapidly evolving field offering substantial clinical potential. This review examines the molecular mechanisms of intracellular enzymatic systems that utilize single-stranded nucleic acids to downregulate specific RNA targets. The analysis encompasses antisense oligonucleotides and synthetic mimics of small interfering RNA (siRNA), microRNA (miRNA), transfer RNA-derived small RNA (tsRNA), and PIWI-interacting RNA (piRNA), elucidating their intricate interactions with crucial cellular machinery, specifically RNase H1, RNase P, AGO, and PIWI proteins, mediating their biological effects. The functional and structural characteristics of these endonucleases are examined in relation to their mechanisms of action and resultant therapeutic outcomes. This comprehensive analysis illuminates the interactions between single-stranded nucleic acids and their endonuclease partners, covering antisense inhibition pathways as well as RNA interference processes. This field of research has important implications for advancing targeted RNA modulation strategies across various disease contexts.

## 1. Introduction

The investigation of intracellular RNA involvement in disease pathogenesis stands as an essential research direction with significant implications for diagnostic and therapeutic purposes. RNA molecules play a key role in gene regulation and protein synthesis, and their dysregulation is increasingly recognized as a driving factor in the pathogenesis of various diseases, including cancer, cardiovascular disorders, neurodegenerative and hereditary conditions, immune disfunctions and viral infections (for reviews see [1,2,3,4,5,6,7]). In addition, RNA dysregulation is strongly associated with aging-related processes such as cellular senescence [8,9] and trained immunity reprogramming [10]. Aberrant RNA processing, stability, localization, and modification contribute to the disruption of cellular homeostasis and the progression of disease. Therapeutic targeting of dysfunctional RNAs offers a promising strategy for the development of innovative homeostatic modulators and therapeutics.

Nucleic acid-based therapeutics have been of significant interest to researchers for several decades as specific regulators of various pathological conditions at the molecular level. In most cases, the use of therapeutic nucleic acids (TNAs) relies on the recruitment of endogenous enzymatic systems, namely RNase H1, RNase P, proteins of the AGO family (AGO and PIWI subfamilies).

Intracellular ribonucleases RNase H1 and RNase P play pivotal roles in fundamental cellular processes of replication and transcription [11,12]. Notably, besides their physiological functions, these enzymes have demonstrated significant potential as effectors in antisense technology [13]. This technology is based on the application of synthetic antisense oligonucleotides (ASOs), whose therapeutic action is executed through sequence-specific binding to a pathological RNA target, followed by recruitment of RNase H1 or RNase P, leading to endonucleolytic degradation of the target molecule. The functional role of ASOs is not limited to mRNA inhibition. Oligonucleotide sequences have been developed with varying mechanisms of action depending on the location of RNA-target: correction of splicing leading to the formation of functional protein variants (binding to the pre-mRNA target at intron-exon junctions or splicing-related sequences and modulating the inclusion or exclusion of specific exons) [14]; activation of protein translation (binding to open reading frames or translation-inhibiting elements in the 5′-untranslated region) [15,16].

The AGO and PIWI protein subfamilies represent two major classes of endoribonucleases that play crucial roles in post-transcriptional gene regulation. AGO proteins are central effectors of the RNA interference (RNAi) pathway, facilitating the cleavage or translational repression of complementary mRNA targets through the formation of RNA-induced silencing complexes (RISC) [17]. PIWI proteins, while initially characterized as germline-specific enzymes for transposon silencing, possess similar endonuclease capabilities and have recently been shown to operate beyond their canonical roles, mediating post-transcriptional gene regulation [18,19,20,21,22,23]. Both AGO and PIWI proteins execute their regulatory functions through interactions with various classes of small non-coding RNAs. AGO proteins associate with small interfering RNAs (siRNAs) and microRNAs (miRNAs), which are the best-characterized functional partners in the RNAi pathway. Contemporary research demonstrates a growing focus on additional types of small RNAs, particularly transfer RNA-derived small RNAs (tsRNAs), as potential regulatory molecules that interact with AGO proteins to initiate gene silencing [24]. PIWI proteins are traditionally associated with the regulation of transposon activity in germline cells through interactions with PIWI-interacting RNAs (piRNAs) [23]. However, recent investigations have revealed that the PIWI-piRNA complexes are also capable of mediating post-transcriptional regulation through a miRNA-like mechanism [25,26]. RNAi represents a conserved molecular mechanism for gene silencing that is currently being actively investigated as a therapeutic strategy for a wide range of human diseases. Recent years have witnessed significant expansion in research exploring various small non-coding RNAs as potential therapeutic agents. The use of synthetic oligonucleotides mimicking endogenous small RNAs (siRNAs, miRNAs, tsRNAs, piRNAs) in collaboration with AGO and PIWI subfamily proteins has been shown to effectively induce RNAi and represents a rapidly developing therapeutic gene silencing strategy.

Many years of research in the field of TNAs development have led to the creation of several generations of modified oligonucleotide analogs characterized by increased nuclease resistance, improved specificity, and, consequently, increased biological activity. The successful application of TNAs for modulating the activity of intracellular RNA targets has led to the development and clinical approval of drugs based on antisense oligonucleotides and siRNAs highlighting the promise of this research field [13,14,27].

This review focuses on the intracellular enzymatic systems whose activity, combined with TNAs, is utilized by researchers to inhibit the function of intracellular RNA targets. Particular attention is given to the structural and functional characteristics of endonucleases, their role in antisense inhibition and RNAi, and an overview of therapeutic effects mediated by this molecular partnership. Within this review, antisense oligonucleotides, synthetic single-stranded siRNAs, miRNA, tsRNA and piRNA mimics, whose biological effects are realized through intracellular endonucleases RNase H1, RNase P, AGO, and PIWI proteins, are thoroughly analyzed. Despite certain advantages of duplex RNAs, such as structural homology with native siRNA and miRNA molecules and increased nuclease resistance, they also have significant drawbacks related to passenger strands: synthesis complexity, potential off-target effects, and reduced tissue penetration capability. In this regard, single-stranded analogs recruiting intracellular enzymes can be considered more promising therapeutic constructs. Consequently, this work provides a comprehensive analysis of partnership interactions of endonucleases with single-stranded TNAs, highlighting molecular prerequisites for effective RNA-targeting technology.

## 2. RNase H1 as a Key Enzyme Facilitating Biological Activity of Antisense Oligonucleotides

### 2.1. Structure, Mechanism of Action and Physiological Functions of RNase H1

The effectiveness of target RNA suppression by antisense oligonucleotides is based on the recruitment of an intracellular nuclease–RNase H1. Members of the RNase H family were first described in 1969 by Stein and Hausen [28] and present sequence-nonspecific, metal-dependent enzymes belonging to the nucleotidyltransferase superfamily, which recognize RNA-DNA heteroduplexes and catalyze the cleavage of phosphodiester bond in the RNA strand, generating products with 3′-hydroxyl and 5′-phosphate groups and releasing the intact DNA strand [12]. RNase H enzymatic activity has been detected across almost all life forms, from bacteria and archaea to eukaryotes. Proteins with RNase H activity have also been identified in viruses *Retroviridae*, *Hepadnaviridae* or *Caulimoviridae* families [29]. Generally, RNase H functions as a sequence-nonspecific endoribonuclease, but Lee et al. reported that RNase H exhibits exoribonuclease activity in the presence of a 3′-overhanging single-stranded DNA end in an RNA-DNA heteroduplex, processively hydrolyzing RNA in the 5′-3′ direction [30].

Based on amino acid sequences and substrate specificity, enzymes of the RNase H family are classified into two main types: RNase H1 and RNase H2, which have identical structural elements of the catalytic domain but different substrate specificity. Additionally, a third type of enzyme–RNase H3–is distinguished, which has structural affinity with RNase H2 but substrate specificity similar to RNase H1 [12]. Enzymes of the RNase H family play an important role in replication and repair processes. RNA-DNA hybrids can be formed in the cell during various physiological processes, such as RNA primer synthesis on the lagging strand during DNA replication (Okazaki fragments), telomere elongation, R-loop formation during transcription, reverse transcription, and erroneous incorporation of ribonucleotides by DNA polymerases [31]. RNase H1 knockout in mice leads to embryonic lethality [32], while mutations in genes encoding RNase H2 result in the development of autoimmune pathologies such as Aicardi-Goutières syndrome [33,34] or systemic lupus erythematosus [35,36], as well as skin and colorectal cancers in humans [37,38,39]. Beyond participating in these essential cellular processes, RNase H enzymes, particularly RNase H1, are crucial mediators in regulation of intracellular RNAs activity guided by antisense oligonucleotides. Interestingly, although RNase H2 predominates in mammalian cells, only RNase H1 participates in target RNA inactivation by ASOs. This is likely due to the fact that RNase H2 is closely associated with chromatin, whereas RNase H1 is localized in the nucleus, cytoplasm and mitochondria [40,41,42].

**Figure 1:**
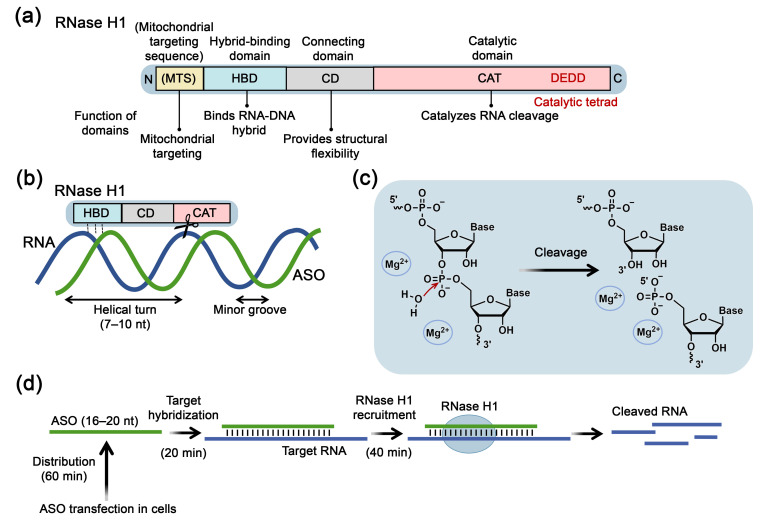
Eukaryotic RNase H1 and its role in target RNA degradation. (**a**) Domain organization of RNase H1. (**b**) Schematic representation of RNase H1 domain interactions with the RNA-DNA heteroduplex modified from [45]. (**c**) Scheme of phosphodiester bond cleavage reaction catalyzed by RNase H enzymes. (**d**) Degradation of target RNA in the complex with antisense oligonucleotide (ASO) via recruitment of RNase H1. The time required for phosphorothioate-ASO distribution, pre-hybridization, and enzyme recruitment stages after ASO transfection into cells indicated in parentheses are according to [46].

RNase H1 (Figure 1) in eukaryotes consists of an N-terminal hybrid-binding domain (HBD) (approximately 50 amino acids (a. a.)) and a C-terminal catalytic domain (CAT) (approximately 150 a. a.), which are connected by a flexible connecting domain (CD) (64 a. a.) that provides spatial mobility of the C- and N-termini necessary for the functional activity of RNase H1 [43]. RNase H1 localized in mitochondria may contain an additional N-terminal sequence necessary for mitochondrial recognition–mitochondrial targeting sequence (MTS) [12,44].

The catalytic domain of RNase H1 includes 4 conserved amino acid residues, comprising aspartic and glutamic acids, which form the DEDD tetrad of the active site (Figure 1a). Mediated by critical lysine and tryptophan residues, the HBD of RNase H1 preferentially binds to RNA-DNA heteroduplexes but can also recognize double-stranded (ds) RNA and DNA, though its affinity for these substrates is reduced by 25- and 100-fold, respectively, without cleavage [47]. RNase H1 covers one helical turn of the DNA/RNA duplex, positioning the catalytic domain directly opposite the minor groove, and catalysis requires the presence of the 2′-OH group of the RNA strand [43] (Figure 1b). The HBD of eukaryotic RNase H1 provides a characteristic substrate cleavage pattern, wherein multiple hydrolysis events occur following a single HBD-mediated binding of RNase H1 to the substrate, resulting in the formation of short RNA fragments that readily dissociate from the complex with DNA [47]. For catalytic hydrolysis of RNA within an RNA-DNA heteroduplex by RNase H1, at least four consecutive ribonucleotides are required. The enzyme binds to the RNA-DNA heteroduplex, exhibiting A-form helix parameters, via HBD, resulting in significant conformational changes in the substrate: the major groove of the duplex expands from 4.2 Å to 12.5 Å, approaching B-form DNA parameters [48]. These structural alterations facilitate the catalytic RNA cleavage occurring at a distance of 7–10 nucleotides from the 5′-end of RNA (one helical turn) (Figure 1b).

The reaction scheme for RNA cleavage by RNase H enzymes is shown in Figure 1c. It involves nucleophilic attack by a deprotonated water molecule on the RNA phosphate, forming a pentavalent intermediate, followed by protonation of the 3′-leaving group, cleavage of the phosphodiester bond, and formation of 5′-phosphate and 3′-hydroxyl products (Figure 1c) [12]. Catalysis requires divalent metal ions, preferably Mg^2+^, coordinated by negatively charged residues (aspartic and glutamic acids) in the RNase H active site, facilitating nucleophile activation and enzyme-substrate destabilization. Researchers have not yet reached a consensus regarding the number of metal ions required for the catalysis by RNase H. Currently, three catalytic models involving one, two, or three metal ions have been proposed [49]. Furthermore, it has been shown that monovalent K^+^ ions are also necessary for coordinating the nucleophile and substrate to facilitate RNase H enzymatic activity [50]. The optimal conditions for RNase H1 activity are 1 mM Mg^2+^ and pH 7–8 [51]. It is also known that hydrolysis can be supported by Mn^2+^ ions [52] and inhibited by Ca^2+^ ions [53].

### 2.2. RNase H1 as an Enzymatic Effector in Antisense Oligonucleotide-Based Therapeutics

#### 2.2.1. Kinetic Parameters of RNase H1-Mediated Target RNA Degradation

The concept of sequence-specific gene expression downregulation using antisense oligonucleotides was proposed in the early 1970s [54,55,56], with initial experimental validation obtained in cell-free systems [57]. These findings laid the foundation for the development of ASO technology and initiated extensive research into the therapeutic potential of ASOs for treating cancers, viral infections, inflammatory processes, blood disorders, and cardiovascular diseases [27,45,58,59]. Depending on the mechanism of action, ASOs are generally classified into three types, including RNase H1-activating, steric-blocking and splice-switching oligonucleotides. In this review, particular focus will be given to RNase H1-activating ASOs, which induce target RNA degradation by recruiting RNase H1.

The molecular pharmacology of ASO/RNase H1 interaction exhibits relatively slow kinetics. According to studies performed by S. Crooke laboratory using phosphorothioate-ASOs (PS-ASOs), it was demonstrated that target RNA reduction occurs approximately two hours after transfection of ASOs into cells (Figure 1d). This period includes 60 min for pre-hybridization stage, involving intracellular ASO distribution, achieving effective concentrations at the target RNA site, 20 min for hybridization to the target sequence, and 40 min for recruitment and cleavage of RNA by RNase H1 (Figure 1d). RNase H1-mediated RNA cleavage occurs 2–4 times faster than the intrinsic degradation rate of cellular RNA [46,60]. During the pre-hybridization stage, the presence of proteins capable of interacting with ASOs can influence the efficiency of RNase H1-mediated antisense action. Many of these proteins contain nucleic acid binding domains or serve as chaperones. The mechanisms by which such proteins influence ASO activity can be diverse. Some proteins, like Ku70/Ku80, hspA8, hnRNP, and P54nrb, inhibit ASO activity by competing with RNase H1 for binding with RNA-ASO heteroduplex [61]. Conversely, proteins such as TCP1, La/SSB, NPM1, ANXA2, VARS, and PC4 enhance ASO activity by altering their subcellular localization [62]. Wu and colleagues identified the mitochondrial protein P32, which binds specifically to the hybrid-binding domain of RNase H1 in a 1:1 ratio increasing RNA cleavage efficiency by reducing enzyme-substrate affinity and accelerating reaction turnover [63]. Zhang et al. revealed that the nucleolar proteins acetyltransferase NAT10 and RNA helicase DDX21 interact with RNase H1 and increase the rate of RNA cleavage in heteroduplexes with PS-ASOs, particularly during the initial 6 h [64]. Although the exact mechanisms are unclear, authors suggest that these proteins may bind to RNase H1 and alter its conformation, providing an increase in its catalytic activity or these proteins may facilitate the dissociation of ASO and RNA after cleavage, and/or promote RNase H1 binding to the ASO-RNA heteroduplex, potentially due to their helicase domains [64].

#### 2.2.2. Chemical Modifications of Antisense Oligonucleotides Compatible with RNase H1 Activity

The rapid degradation of natural oligodeoxyribonucleotides by nucleases in vivo necessitates the introduction of various chemical modifications into ASOs to enhance their stability. These strategic changes not only increase nuclease resistance but can also improve hybridization properties, enhancing the biological efficiency of ASOs. Despite certain advantages, most chemically modified oligonucleotides in heteroduplexes with RNA hinder the recognition and degradation of target RNAs by RNase H1.

**Figure 2:**
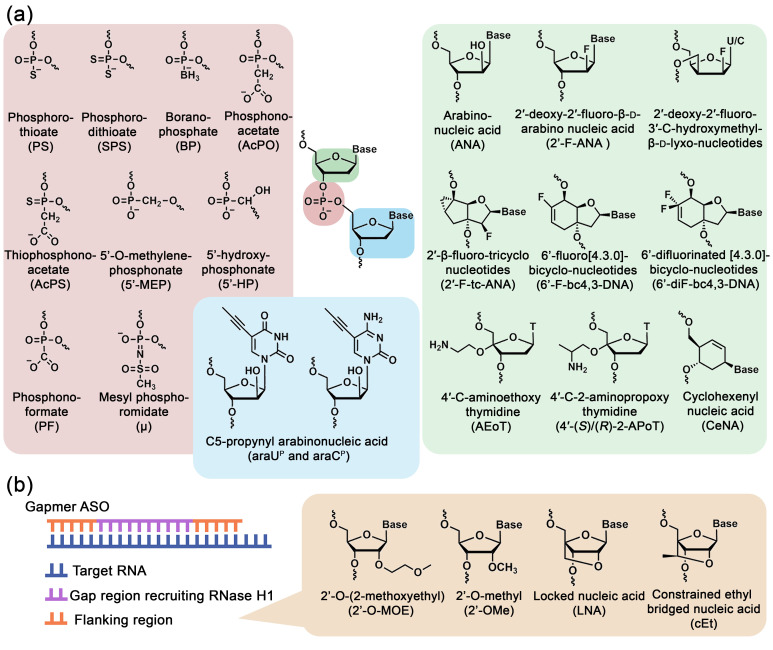
Chemical modifications compatible with RNase H1 activity and structure of gapmer-type antisense oligonucleotides. (**a**) Examples of chemical modifications that, as part of ASOs, promoting heteroduplex formation with target RNA recognizable by RNase H1. Modifications of the phosphodiester backbone are highlighted in red, sugar moiety modifications in green, and modifications of nucleobases and sugar in blue. (**b**) Structure of gapmer ASO, consisting of a central region recognized by RNase H1, and flanking regions containing modified oligonucleotides (orange frame) enhancing target-binding efficiency and/or nuclease resistance. The structure of FDA-approved gapmer ASOs, such as mipomersen, inotersen, volanesorsen and tofersen, is characterized by a fully PS-modified backbone with five 2′-O-MOE modifications flanking each terminus. Cytosines within these molecules are typically methylated at the 5′-position, enhancing their stability and affinity for target RNA.

The catalytic center of RNase H1 is sensitive even to minimal changes in the helical geometry of the duplex. Several essential requirements for the ASO-RNA heteroduplex have been identified to ensure effective RNase H1 recruitment: (1) the ASO must have sufficient flexibility to maintain the ASO-RNA heteroduplex conformation required for RNase H1-mediated RNA cleavage; (2) the helical structure of the ASO-RNA heteroduplex should have a minor groove width close to that of natural RNA-DNA duplexes–8.9–10.5 Å, compared, for example, to DNA-DNA duplex with a minor groove width of 3–7.4 Å; (3) the minor groove geometry must remain unchanged to maintain the steric accessibility of the 2′-OH of RNA and ensure optimal hydration environment [65,66]. In ASO-RNA heteroduplex, the RNA 2′-OH is a key participant in RNase H1-dependent catalytic cleavage. Consequently, the introduction of various modifications affecting the 2′-position of ribose or nucleotide bases in the nucleic acid strand, significantly altering duplex helical geometry, restrict RNase H1 cleavage activity [65]. Despite the sensitivity of RNase H1 to the structural organization of ASO-RNA heteroduplexes, a number of modifications compatible with RNase H1 activity have been developed (Figure 2a).

Studies have shown that chemical modifications of ASOs that exert the least restrictive influence on the ability to recruit RNase H1 include internucleotidic bond modifications, in which one or two non-bridging oxygen atoms of the phosphate group are replaced by other atoms or chemical groups. The phosphorothioate (PS) modification, which involves replacing one of the oxygen atoms of the internucleotidic phosphate group with a sulfur atom, was the first and remains the most common ASO modification, providing high nuclease resistance and the ability to form heteroduplexes in which RNA undergoes cleavage by RNase H1 (Figure 2a; Table 1) [67,68]. Additional favorable characteristics of PS modification include increased affinity for plasma proteins, improving the pharmacokinetics of oligonucleotides, and facilitating cellular uptake and biodistribution. Despite a number of advantages, there are also disadvantages of PS-oligonucleotides, such as limited efficiency and specificity of binding to target RNAs, reduced ability to recruit RNase H1 compared to unmodified analogs, as well as relatively high toxicity due to nonspecific interactions with cellular proteins [67,68]. Nevertheless, despite certain imperfections, PS modification is currently widely used in clinically approved ASO-based drugs.

In addition to phosphorothioate (PS) modifications, phosphate group modifications that maintain RNase H1 activity also include phosphorodithioate (SPS) [69,70], boranophosphates [71,72,73], phosphonoacetate (AcPO) [74] and thiophosphonoacetate (AcPS) [74], which enhance nuclease resistance, but exhibit lower binding efficiency with complementary RNA compared to unmodified ASOs (Figure 2a; Table 1). Among phosphate group modifications, 5′-O-methylphosphonate (5′-MEP) [75], 5′-hydroxyphosphonate (5′-HP) [75] and phosphonoformate (PF) [76] modifications are prominent, as they demonstrated increased thermodynamic stability of the duplex compared to unmodified analogs (Figure 2a; Table 1).

**Table 1:** Characteristics of oligonucleotide modifications compatible with RNase H1 activity.

Modification	Nuclease Resistance ^1^	Duplex Stability ^2^	Studies in Biological Systems	Reference
Phosphorothioate (PS)	+	−	Cell culture/in vivo	[67,77]
Phosphorodithioate (SPS)	+++	−	n.s.	[69,70]
Phosphonoacetate (AcPO)	+++	−	n.s.	[74]
Thiophosphonoacetate (AcPS)	+++	−	n.s.	[74]
Phosphonoformate (PF)	+++	+	n.s.	[76]
Boranophosphate	++	−	n.s.	[71,72,73]
Mesyl-(methanesulfonyl)-phosphoramidate (μ)	+++	−	Cell culture/in vivo	[78,79,80,81,82]
5′-O-methylenephosphonate (5′-MEP)	n.s.	+	n.s.	[75]
5′-hydroxyphosphonate (5′-HP)	n.s.	+	n.s.	[75]
Arabinonucleic acid (ANA)	+	−	n.s.	[83,84]
2′-deoxy-2′-fluoro-β-d-arabino nucleic acid (2′-F-ANA or FANA)	++	+	Cell culture/in vivo	[84,85,86,87,88,89,90]
2′-deoxy-2′-fluoro-3′-C-hydroxymethyl-β-d-lyxo-configured pyrimidine nucleotides (U/C)	+	+	Cell culture	[91]
2′-β-fluoro-tricyclo nucleotides (2′-F-tc-ANA)	n.s	+	n.s.	[92]
6′-fluoro[4.3.0]bicyclo-nucleotides (6′-F-bc4,3-DNA)	n.s.	− −	n.s.	[93]
6′-difluorinated[4.3.0]bicyclo-nucleotides (6′-diF-bc4,3-DNA)	n.s.	− −	n.s.	[94]
4′-C-aminoethoxy thymidine (AEoT)	+++	+	Cell culture	[95]
4′-C-2-aminopropoxy thymidine in *S*- and *R*-configuration (4′-(*S*)-2-APoT and 4′-(*R*)-2-APoT)	+++	−	n.s.	[96]
Cyclohexenyl nucleic acid (CeNA)	+++	+	n.s.	[97,98]
C5-propynyl arabinouridine (araU^P^) and arabinocytidine (araC^P^)	+++	+	n.s.	[99]

^1^ The estimated assessment of nuclease resistance of modified oligonucleotides, represented as mediate, enhanced, or superior resistance, indicated by +, ++, or +++, respectively. ^2^ Duplex stability between modified DNA and RNA, where “–” denotes a decrease in Tm of less than 3 °C per modification, “– –” a decrease of less than 5 °C, and “+” indicates an increase in Tm. “n.s.” indicates that corresponding studies were not performed.

Currently, the newly developed phosphate group modification, compatible with RNase H1 activity is mesyl-(methanesulfonyl)-phosphoramidate (mesyl or μ). Depending on the length and type of RNA target (mRNA or miRNA), mesyl oligonucleotides demonstrate similar or slightly reduced hybridization properties compared to natural DNA. Notably, fully mesyl-modified oligonucleotides demonstrate unprecedented resistance to nucleases, surpassing the stability of PS-oligonucleotides while maintaining functional activity [78,81,82,100] (Figure 2a; Table 1).

Most chemical modifications of the sugar moiety and nucleobases generally do not support RNase H1 activity; however, there are some variants of ribose modifications and base analogs that, under certain conditions, do not impede the interaction of RNase H1 with the formed heteroduplex. One of the most well-known examples of 2′-position ribose modifications that do not inhibit RNase H1 activity are modifications based on arabinonucleic acid [83,84], which additionally increase affinity to complementary RNA and enhance nuclease stability of ASOs compared to unmodified analogs. These modifications include 2′-deoxy-2′-fluoro-β-d-arabino nucleic acid (2′-F-ANA or FANA) [84,85,86], and 2′-deoxy-2′-fluoro-3′-C-hydroxymethyl-β-d-lyxo-configured pyrimidine nucleotides (U/C) [91] (Figure 2a; Table 1). Although fully modified FANA does not support RNase H1 activity, its incorporation into chimeric PS-oligonucleotides with alternating FANA and DNA nucleotides, or in oligonucleotides containing a central DNA region with flanking FANA regions, provides effective RNase H1-mediated cleavage of the target RNA, comparable to PS-oligonucleotide [86]. Moreover, a unique feature of FANA is its ability to penetrate cells without the use of delivery agents [101]. Modification with a propynyl group at the C5 position of the arabinouridine or arabinocytidine nucleotide bases (C5-propynyl arabinouridine (araU^P^) and (araC^P^)) preserve the RNase H1-activating capability of ASOs when incorporated into the central region flanked by DNA or 2′-O-MOE nucleotides [99] (Figure 2a; Table 1).

Nucleotide analogs, retaining RNase H1 activity, containing fluorine atoms in various positions of the tri- and bi-cyclic sugar backbone structures are being actively developed–2′-β-fluoro-tricyclo nucleotides (2′-F-tc-ANA) [92], 6′-fluoro[4.3.0]bicyclo-nucleotides (6′-F-bc4,3-DNA) [93], 6′-difluorinated[4.3.0]bicyclo-nucleotides (6′-diF-bc4,3-DNA) [94] (Figure 2a; Table 1). It has been shown that oligonucleotides containing 5 consecutive modified nucleotides flanked by DNA bases support RNase H1-mediated cleavage of chimeric 2′-O-Me RNA containing ribonucleotides opposite the modified region [94].

Examples of nucleotide analogs that do not impede RNase H1 activity when selectively incorporated within oligonucleotides, while simultaneously enhancing serum stability, include modifications of the 4′-position of deoxyribose such as 4′-C-aminoethoxy thymidine (AEoT) [95] and 4′-C-2-aminopropoxy thymidine in *S*- and *R*-configuration (4′-(*S*)-2-APoT and 4′-(*R*)-2-APoT) [96], as well as nucleotide analogs with a six-membered ring, such as cyclohexenyl nucleic acid (CeNA) [97,98,102] (Figure 2a; Table 1).

Despite the broad diversity of chemical modifications capable of operating in partnership with RNase H1, only a limited number is currently employed in the design of RNA-targeting antisense oligonucleotides for use in biological systems in vitro and in vivo (Table 1). As previously mentioned, PS modification has emerged as the predominant approach and are incorporated into virtually all clinically approved RNase H1-recruiting ASOs, establishing them as the gold standard in this therapeutic modality [13,27].

Among recently developed promising analogs, mesyl-phosphoramidate modifications warrant particular attention due to their high efficiency in suppressing mRNA [81,82], as well as oncogenic long non-coding RNA (lncRNA) [103] and miRNAs, with demonstrated success in murine tumor models [78,79,80]. FANA modifications also represent a significant advancement and are actively used for incorporation into the terminal regions of oligonucleotides containing a central DNA region to suppress therapeutically significant targets both in vitro and in vivo [86,87,88,89,90,101]. These modifications not only provide nuclease resistance and facilitate RNase H1 recruitment but also enhance cellular uptake without the necessity of transfection agents.

Among recently developed ribose analogs, biological activity in target mRNA suppression has been demonstrated primarily for oligonucleotides incorporating 2′-deoxy-2′-fluoro-3′-C-hydroxymethyl-β-d-lyxo-configured pyrimidine nucleotides (U/C) and 4′-C-aminoethoxy thymidine (AEoT) [91,95]. While investigations of other RNase H1-recruiting modifications remain largely limited to physicochemical characterization, they nonetheless possess considerable potential for integration into next-generation oligonucleotide-based technologies.

The most common and well-established structural format of RNase H1-recruiting ASOs is the gapmer design. Gapmers are chimeric oligonucleotides that contain a central block (6–10 nucleotides) consisting of DNA and PS monomers capable of inducing RNase H1 cleavage, flanked by modified nucleotides (2–5 nucleotides), such as 2′-methoxyethyl (2′-O-MOE), locked nucleic acids (LNA), constrained ethyl nucleic acids (cEt), or 2′-O-methyl (2′-OMe), which are not identified by RNase H1 but enhance ASO binding efficiency to RNA and in some cases additionally increase their nuclease stability (Figure 2b) [27,45]. Gapmer architectures have emerged as the predominant therapeutic platform due to their mechanistically superior balance of target binding affinity, nuclease resistance, and RNase H1 activation efficiency. Extensive research efforts continue to optimize gapmer architectures through refinement of gap length and flanking modifications to further maximize therapeutic efficacy.

#### 2.2.3. Therapeutic Outcomes of RNase H1-Mediated Antisense Activity

Currently, there is substantial interest in studying antisense oligonucleotides targeted at suppressing therapeutically significant RNA targets. Among antisense constructs utilizing RNase H1 activity, a clear preference is given to gapmer structures, which represent the predominant majority among clinically approved antisense drugs and their widespread use in on-going clinical development programs. This design preference has become nearly universal in the development of RNase H1-mediated therapeutics, targeting diverse therapeutic RNA targets, ranging from protein-coding mRNAs to various non-coding RNAs, with only rare exceptions favoring alternative designs.

RNase H1-activating antisense oligonucleotides (ASOs) are widely used to suppress protein-coding mRNAs associated with hereditary diseases, representing the most advanced therapeutic application in the oligonucleotide field. Several notable examples have received clinical approval, including 2′-O-MOE gapmers (Figure 2b) targeting apolipoprotein B-100 (*ApoB-100*) mRNA for the treatment of homozygous familial hypercholesterolemia (mipomersen), transthyretin (*TTR*) mRNA in hereditary transthyretin-mediated amyloidosis (inotersen and eplontersen), apolipoprotein CIII (*Apo-CIII*) mRNA in familial chylomicronemia syndrome (volanesorsen and olezarsen), and superoxide dismutase 1 (*SOD1*) mRNA in amyotrophic lateral sclerosis (tofersen) [104,105,106,107,108,109].

Gapmer mRNA-targeted oligonucleotides have also demonstrated high efficiency against a wide range of viral infections. LNA-gapmers were efficient in suppressing replication of various viruses by targeting critical regions such as the Internal ribosome entry site (IRES) of hepatitis C virus [110], the host gene Niemann-Pick C1 (*NPC1*) essential for Ebola virus entry [111,112], and the 3′-untranslated region (3′-UTR) region of viral RNA in Japanese encephalitis virus [113]. FANA-gapmers targeting highly conserved regions of human immunodeficiency virus 1 (HIV-1) genome (U5, tat/rev, and dimerization initiation site) have been shown to suppress expression of viral RNA in primary human cells [87]. In response to the COVID-19 pandemic, the development of gapmers targeting various regions of severe acute respiratory syndrome coronavirus 2 (SARS-CoV-2) RNA has progressed rapidly. Studies have demonstrated that various types of gapmers, including LNA- and cEt-gapmers, can inhibit virus replication in vitro and in vivo [114,115,116,117].

Recently, the spectrum of therapeutic targets for antisense therapy was significantly expanded. In addition to traditional protein-coding mRNAs, researchers are increasingly focused on classes of non-coding RNA, particularly those implicated in cancer development. For example, LNA-gapmers was developed against the primary transcript of the miR-17-92 cluster [118], while 2′-O-MOE gapmer conjugated with tocopherol was focused on supression of MIR17HG precursor RNA [119]. One of the rare examples of effective application of oligonucleotides possessing a homogeneous rather than chimeric/gapmer structure is mesyl phosphoramidate-modified ASOs, which demonstrated significant efficiency in the inhibition of mature oncogenic miRNAs miR-21, miR-17, and miR-155 [79,80]. LNA-gapmers were designed to silence circular RNA (circRNA) by targeting their unique backsplice-junction sequence [120], and LNA-gapmers was also designed to suppress piRNA piR-32877 [121]. For lncRNAs, examples include LNA-gapmer targeting PURPL in hepatocellular carcinoma [122] or PVT1 in squamous cell skin cancer [123], and cEt- or LNA-gapmers directed against MALAT1 [124,125]. The main effects observed following the use of RNase H1-activating ASOs targeting ncRNAs included suppression of tumor cell proliferation and migration, as well as induction of apoptosis in vitro [79,80,118,119,120,121,122,123,124,125] along with inhibition of tumor growth and metastasis in vivo [79,80,118,119,123,124,125].

## 3. RNase P-Mediated RNA Inhibition Using External Guide Sequences

**Figure 3:**
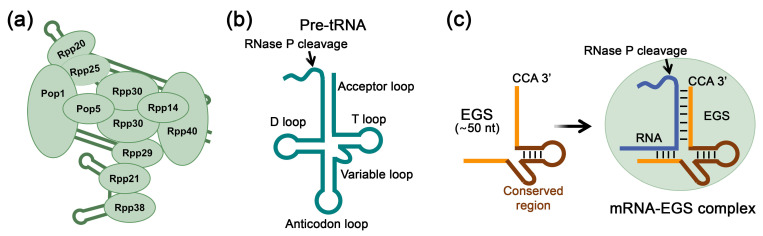
Structural and functional characteristics of RNase P-mediated cleavage of target RNA using external guide sequences (EGS). (**a**) Structural organization of human RNase P, consisting of catalytic RNA component (H1 RNA) (green line) and ten auxiliary protein subunits (green circles). (**b**) Structure of precursor tRNA (pre-tRNA), a natural substrate of RNase P. (**c**) Complex formed by EGS and target mRNA, structurally mimicking pre-tRNA and specifically recognized and cleaved by RNase P.

A promising approach to nucleic acid-based gene therapy involves utilizing the ribonuclease activity of the endogenous enzyme RNase P. Human RNase P is a ribonucleoprotein complex consisting of a catalytic RNA component (H1 RNA) and an auxiliary protein component (Figure 3a). The enzyme plays a key role in processing transfer RNAs (tRNAs) by specifically cleaving the 5′-leader sequence of precursor tRNAs (pre-tRNAs) (Figure 3b) [11,126,127]. For selective suppression of gene expression utilizing the activity of endogenous RNase P, an external guide sequence (EGS) is employed, which is a short synthetic RNA sequence approximately 50 nucleotides in length that mimics a pre-tRNA fragment [128,129] (Figure 3c). This molecule contains two essential structural regions: a conserved region modeling the topology of the tRNA precursor recognized by RNase P, and a specific region complementary to the target mRNA. The therapeutic effect is realized by the formation of a complex between EGS and target mRNA, structurally mimicking the natural RNase P substrate, resulting in specific cleavage of the target mRNA by the enzyme and reducing its expression levels.

The first application of EGS-RNase P technology for selective gene expression suppression was demonstrated in Sidney Altman laboratory [128,129]. Since then, this approach has been actively developed for therapeutic applications. Initial experimental efforts focused primarily on bacterial systems and demonstrated that EGS constructs could successfully inhibit the expression of essential genes and impair viability in a range of clinically relevant pathogens, including *Escherichia coli*, *Salmonella typhimurium*, *Klebsiella pneumoniae*, *Mycobacterium* *spp.*, *Staphylococcus aureus*, and the protozoan parasite *Plasmodium falciparum*, the causative agent of severe malaria [130,131]. A recent example highlighting the antibacterial potential of EGS involves *Acinetobacter baumannii*, which causes severe nosocomial infections. In this study, DNA-EGS and 2′-OMe EGS-oligonucleotides conjugated with oligo(N-methyl-pyrrole) unassistedly penetrated bacterial cells. Effective *ftsZ* and *gyrA* mRNAs cleavage mediated by RNase P was achieved [132]. EGS technology has also been widely explored for inhibiting human viral gene expression, including human influenza virus, human cytomegalovirus (HCMV), herpes simplex virus type 1 and 2 (HSV-1, HSV-2), human immunodeficiency virus (HIV), hepatitis B virus (HBV) and Kaposi sarcoma-associated herpesvirus, as comprehensively reviewed in [131]. Recent studies have explored the application of EGS oligonucleotides to target critical viral transcripts, the major capsid protein (*MCP*) mRNA of HCMV [133], mRNA encoding the HSV-2 ssDNA-binding protein ICP8 of HSV-2, which is necessary for viral replication [134] and the human CC-chemokine receptor 5 (*CCR5*) mRNA encoding a co-receptor essential for HIV entry [135]. These EGSs demonstrated high efficacy, achieving up to a 98% reduction in target protein levels and suppressing viral replication by several orders. For instance, *MCP* expression in HCMV-infected cells was reduced by 98% with a corresponding 10,000-fold decrease in viral titer [133]; EGSs targeting HSV-2 *ICP8* mRNA led to an 85% reduction in protein levels and a 3000-fold inhibition of virus replication [134]; and *CCR5*-targeting EGSs suppressed mRNA level by 98% and reduced intracellular HIV RNA level by more than 900-fold in vitro [135].

## 4. Argonaute Family Proteins for Target RNA Inhibition

Argonaute family proteins play a pivotal role in gene regulation through RNAi, involving small RNAs within the RISC. These proteins have been identified across nearly all eukaryotic organisms, as well as in bacteria and archaea [136]. The unifying structural element of all proteins in this family is the catalytic PIWI domain, which confers endonuclease activity. Based on sequence identity, eukaryotic Argonaute proteins are categorized into two subfamilies: AGO and PIWI [137]. AGO subfamily proteins are ubiquitously expressed in most tissue types and interact with miRNAs and siRNAs, thereby playing a central role in post-transcriptional gene regulation. Meanwhile, PIWI subfamily proteins, predominantly expressed in germline cells, bind to piRNAs and are involved in the suppression of transposon activity.

### 4.1. Structure, Mechanism of Action, and Key Functions of AGO Proteins

**Figure 4:**
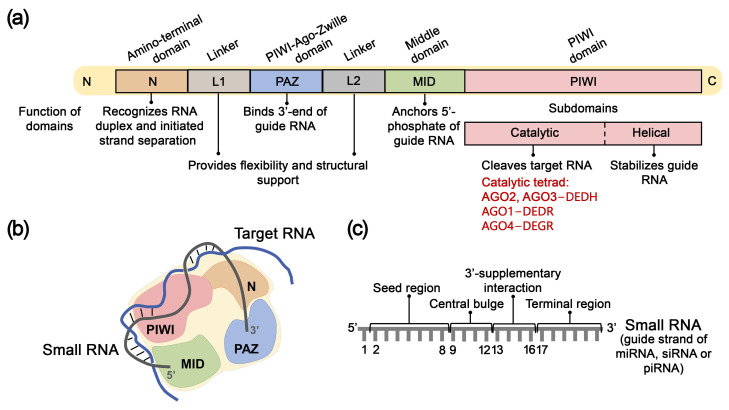
Domain organization and functional structure of human AGO family proteins. (**a**) Schematic representation of AGO/PIWI protein domain organization with description of their main functions. (**b**) Model of AGO protein interaction with the small RNA and target RNA heteroduplex. (**c**) Functional regions of the small RNA guide strand upon itsincorporation into AGO protein.

In humans, the AGO protein subfamily comprises four paralogs with 80% amino acid sequence homology: AGO1, AGO2, AGO3, and AGO4. AGO proteins are characterized by highly conserved structural architecture and consist of four primary domains: N-domain, PAZ domain, MID domain, and PIWI domain [138] (Figure 4a). The N-domain (amino-terminal domain) recognizes miRNA or siRNA duplexes and initiates the separation of guide and passenger RNA strands, accompanied by the assembly of RISC. Subsequently, the AGO protein anchors the guide RNA strand through specific binding of the 3′-end of small RNA by the PAZ domain (PIWI-Argonaute-Zwille domain) and fixation of the 5′-phosphate by the MID domain (Middle domain) (Figure 4a,b). The C-terminal PIWI domain is structurally similar to the catalytic domain of RNase H and plays a crucial role in cleaving target mRNA complementary to small RNA. The PIWI domain contains two subdomains: the PIWI-catalytic and PIWI-helical subdomains (Figure 4a). The PIWI-helical subdomain, in conjunction with the MID domain, forms a composite binding site for the 5′-monophosphate of the guide RNA strand (Figure 4a,b). The N and PAZ domains, as well as the PAZ and MID domains, are connected by linkers L1 and L2, respectively, which ensure structural stability of RISC (Figure 4a). Additionally, AGO proteins contain an unstructured N-terminal region (“beam”) preceding the N-domain, which provides structural stability by connecting the N-domain with linkers L1, L2, and the PIWI domain [138,139,140]. AGO1, AGO3, and AGO4 proteins contain unique structural elements, such as the conserved segment 7, AGO3-specific insertion, and AGO4-specific insertion, respectively, which participate in target recognition or binding to guide RNA. AGO2 and AGO3 proteins share an identical catalytic tetrad of amino acid residues within the PIWI domain—DEDH whereas in AGO1 and AGO4 proteins, this tetrad is represented by amino acids DEDR and DEGR, respectively [138].

It is generally accepted that miRNAs are incorporated into the four AGO paralogs randomly, depending on the miRNA expression profile within the cell [138]. However, studies have demonstrated preferential binding of certain miRNAs to specific AGO proteins. For instance, AGO2 binds miR-342-3p 19 times more frequently than AGO3, whereas AGO3 associates with miR-629-3p and miR-92b-3p 15 and 12 times more actively than AGO2, respectively [138]. Additionally, miR-3191-5p has been detected exclusively in complexes with AGO2 or AGO4 [141]. Nevertheless, the cellular systems regulating this process remain undetermined so far.

Upon incorporation into AGO, each nucleotide of the guide strand of siRNA or miRNA assumes a specific function depending on its position. The sequence of a small RNA comprises five functional regions (Figure 4c): the 5′-terminal nucleotide, which interacts with the MID domain of AGO and does not participate in target recognition; the seed region (nucleotides 2–8); the central region (nucleotides 9–12); the 3′-supplementary region (nucleotides 13–16); and the 3′-terminal region (tail; nucleotides 17–23), which 3′-terminal nucleotide is anchored in the PAZ domain of the AGO protein [140].

Initially, the RNA target interacts with AGO-bound small RNA to form complementary base pairs with its seed region, after which base pairing extends into the 3′-supplementary region. Subsequently, the miRNA strand undergoes reorganization in its terminal region, initiating complete or partial base pairing in the central loop region. Following the formation of a cleavage-competent conformation of the miRNA-mRNA duplex, AGO executes cleavage of the target mRNA opposite the bond between the 10^th^ and 11^th^ nucleotides of the miRNA. The catalytic activity of AGO requires divalent metal ions (Mg^2+^, Mn^2+^, Ni^2+^, Co^2+^) [142,143].

In 2004, two research groups demonstrated that despite AGO2 and AGO3 sharing an identical catalytic tetrad DEDH only AGO2 was capable of cleaving target mRNA [144,145]. In 2017, Park and colleagues showed that human AGO3 could also exhibit nuclease activity, but only in complex with specific miRNAs. For example, when loaded with miR-20a AGO3 cleaved complementary targets, albeit at a lower rate than AGO2 [146]. Subsequently, it was discovered that human AGO3 could demonstrate nuclease activity when loaded with certain tyRNAs (tiny RNAs), short RNAs approximately 14 nucleotides in length that regulate genes and protect the genome from mobile genetic elements [147]. tyRNAs represent RNAs less than 19 nucleotides long that originate from various types of intracellular RNAs: from tRNAs through cleavage by angiogenin, Dicer, or other RNases; from AGO-associated mature miRNA molecules via exonucleolytic removal of nucleotides from the 3′-end; from loop-miRs, which are loop regions of miRNA precursors that constitute “byproducts” formed during the cleavage of hairpin miRNA precursors by Dicer protein; as well as from viral miRNAs [148,149]. It is generally accepted that AGO1 is incapable of cleaving RNA targets [150,151]; however, Wang and colleagues demonstrated AGO1 ability to cleave the passenger RNA strand during RISC assembly [152].

AGO proteins are localized not only in the cytoplasm but also in the nucleus, where they can perform additional non-canonical functions, such as regulation of transcription through interaction with RNA polymerase II or small RNAs, participation in alternative splicing via histone methylation, as well as maintenance of genome integrity through involvement in the repair of DNA double-strand breaks [153,154].

### 4.2. AGO-Mediated RNA Inhibition Using Single-Stranded Oligonucleotides

RNAi mediated by AGO family proteins now represents a rapidly evolving therapeutic strategy for numerous human diseases. The specificity of RISC function is determined by small non-coding RNAs, such as siRNAs and miRNAs. siRNAs are double-stranded RNA molecules 20–25 nucleotides in length that constitute an evolutionarily conserved element of antiviral defense across a broad range of eukaryotic organisms [155,156]. miRNAs represent endogenously expressed double-stranded short non-coding RNA molecules 20–23 nucleotides in length that are involved in the regulation of genes controlling key biological processes within the cell, such as differentiation, proliferation, apoptosis and others [157]. Despite differences in their origin and functional roles, siRNA and miRNA biogenesis occur via the same intracellular mechanism, including stepwise catalytic processing of long hairpin precursors to form short double-stranded siRNA and miRNA molecules [158]. The maturation of these molecules culminates in the formation of a complex between a single-stranded guide RNA and AGO family proteins, ultimately resulting in RISC assembly. Under the action of siRNAs, post-transcriptional regulation is achieved through perfectly complementary binding to target mRNA, followed by AGO2-mediated endonucleolytic cleavage of the target. Regulation by miRNAs may involve several possible scenarios of post-transcriptional silencing, including endonucleolytic cleavage or non-catalytic recruitment of accessory enzymes to execute translational silencing. This process involves elongation factor 4F (eIF4F) to prevent translation initiation, and GW182 protein, which recruits the CCR4-NOT deadenylase complex and facilitates shortening of the poly(A) tail, ultimately leading to mRNA destabilization and degradation [159,160]. As a result of perfectly complementary binding, siRNAs typically target one specific RNA, whereas miRNAs generally form imperfectly complementary complexes with their target mRNAs, thereby controlling the expression of multiple molecules. The use of synthetic oligonucleotides mimicking endogenous siRNAs or miRNAs has been shown to effectively induce RNAi and represents a rapidly developing therapeutic gene silencing modality.

Initial investigations in the field of RNA interference focused on examining the properties of canonical double-stranded synthetic siRNAs and subsequently miRNAs. These studies established the fundamental foundation for developing therapeutic agents aimed at selective gene expression suppression through the RISC pathway. Later, a notable advancement was the discovery that single-stranded analogs (ss-siRNAs and ss-miRNAs) could effectively inhibit the expression of complementary mRNA targets through RNAi mechanisms, which defined a new direction in the development of therapeutic approaches to gene regulation based on single-stranded small RNAs [161,162,163,164,165,166,167,168,169,170,171].

Lima and colleagues [172] demonstrated that the affinity of Dicer and AGO2 to short single-stranded RNAs is comparable to that of classical double-stranded siRNAs. The formation of nucleoprotein complexes involving these single-stranded structures also leads to effective target inactivation via the RNAi pathway. Although in most cases ss-siRNAs exhibit lower or similar efficiency in suppressing target gene expression compared to canonical double-stranded analogs, and demonstrate reduced stability in biological environments [163,166,167,168,169], the application of single-stranded siRNAs offers several substantial advantages: a significantly simpler and more economical production process, rapid formation of functionally active RISC due to reduced processing and loading stages, minimization of side effects due to the absence of passenger strand, and improved pharmacokinetic characteristics. Limitations associated with decreased nuclease resistance can be addressed through rational introduction of chemical modifications into the RNA strand sequence.

#### 4.2.1. Single-Stranded siRNA

In this context, the application of single-stranded siRNAs is considered a promising therapeutic strategy that integrates the best characteristics of existing technologies, combining the simplicity and biological accessibility of single-stranded structures with the unique advantages of recruiting RISC enzymatic activity.

**Figure 5:**
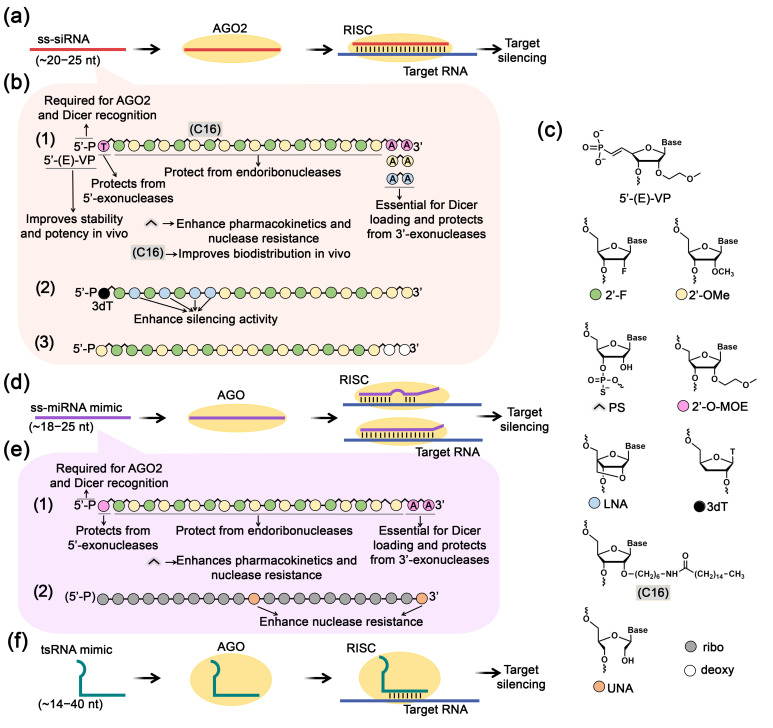
AGO-mediated inhibition of target RNAs using synthetic single-stranded oligonucleotides. (**a**) Scheme of target RNA suppression by single-stranded small interfering RNA (ss-siRNA) involving AGO2 protein. (**b**) Generalized optimized structures of modified ss-siRNAs used for gene suppression as reported in [161,162,164,165,166,167,168] (**1**), [169] (**2**), [170] (**3**). (**c**) Chemical structures of modifications used in the design of the single-stranded oligonucleotides. (**d**) Scheme of target RNA suppression by single-stranded microRNA (ss-miRNA) mimic involving AGO2 protein. (**e**) Chemically modified structures of ss-miRNA mimics used for gene suppression in [163] (**1**) and [171] (**2**). (**f**) Scheme of target RNA suppression by transfer RNA-derived small RNA (tsRNA) involving AGO protein. RISC—RNA-induced silencing complex; 5′-(E)-VP—5′-(E)-vinylphosphonate; 2′-F—2′-fluororibose; 2′-OMe—2′-O-methyl; PS—phosphorothioate; 2′-O-MOE—2′-O-methoxyethyl; LNA—locked nucleic acid; 3dT—3′-deoxythymidine; (C16)−lipophilic linear carbon chain; UNA—unlocked nucleic acid; ribo—ribonucleotide; deoxy—deoxyribonucleotide.

Research aimed at the development of therapeutic ss-siRNAs has elucidated that effective suppression of target mRNA necessitates specific chemical elements in the ss-siRNA architecture (Figure 5a–c). In particular, the presence of a 5′-phosphate is an essential requirement, ensuring recognition and binding of ss-siRNA by AGO and Dicer enzymes. The introduction of metabolically stable phosphate variants that maintain conformational and stereochemical similarity to natural phosphates has proven to be most effective. Another important parameter is the inclusion of an AA dinucleotide at the 3′-end of ss-siRNA. While this element is not critical for interaction with AGO2 protein, it is crucial for binding to Dicer protein, which presumably facilitates molecular transport to RISC [161]. To protect ss-siRNAs preemptively from degradation in biological systems, strategic chemical modifications are employed: protection of 5′ and 3′-terminal regions against exonucleases and modification of the sugar-phosphate backbone to confer resistance against endonuclease activity (Figure 5a–c). To enhance biological properties, the researchers developed ss-siRNAs with the following optimized design (Figure 5b(1)) incorporating various chemical modifications: (I) metabolically stable 5′-phosphate analogs–5′-(E)-vinylphosphonate (5′-(E)-VP) or 5′-methylenephosphonate; (II) 2′-O-MOE-modified 5′-terminal nucleotide for protection against 5′-exonucleases; (III) alternating 2′-fluoro (2′-F) and 2′-OMe ribose modifications that preserve AGO2 cleavage capability, with phosphorothioate internucleotidic bonds for protection against endonucleases and improved pharmacokinetic properties; (IV) 2′-O-MOE-modified adenosine dinucleotide at the 3′-end for protection against 3′-exonucleases; (V) lipophilic C16 modification to enhance tissue biodistribution (Figure 5b(1),c).

Considering these design parameters, several studies leaded by Crooke and Corey have demonstrated the effective use of ss-siRNAs to silence diverse molecular targets including metabolic regulators such as phosphatase and tensin homolog (*PTEN*), coagulation factor VII, and apolipoprotein CIII (*ApoCIII*) in hepatocytes, as well as the mutant *HTT* gene associated with Huntington’s disease (Figure 5b(1),c) [161,162,164,165]. The enhanced ss-siRNA structure substantially improved silencing efficacy, demonstrating a 20-fold increase in target mRNA suppression compared to unmodified variants, extended tissue half-life to approximately 10 days, and sustained therapeutic effect for up to a week in mouse models [161]. For neurodegenerative applications, intraventricular administration facilitated brain-wide suppression of the mutant *HTT* allele, while *ApoCIII*-targeted constructs effectively reduced triglyceride and LDL cholesterol levels in metabolic disease models [162,164,165]. On the basis of the core ss-siRNA design, researchers implemented several additional key enhancements. The integration of a lipophilic C16 linear carbon chain significantly improved cellular penetration and tissue distribution (Figure 5b(1),c) [161,165,173]. Strategic introduction of central mismatches at positions 9,11 or 9,14 generated miRNA-like structures with enhanced allele specificity [164]. Further optimization of the 5′-phosphate region identified 5′-(E)-vinylphosphonate in trans orientation as the optimal modification, balancing metabolic stability with effective AGO2 recruitment capacity (Figure 5b(1),c) [161,162,164,165]. These structural refinements collectively advanced ss-siRNA as a promising platform for targeted gene silencing therapeutics.

Modified ss-siRNAs utilizing a natural 5′-phosphate design demonstrated promising therapeutic potential for targeting pathological repeat expansions in neurological and ocular disorders (Figure 5b(1),c). These constructs effectively targeted the mutant *c9orf72* containing expanded GGGGCC repeats linked to amyotrophic lateral sclerosis and frontotemporal dementia [166]. When fully complementary to the repeat transcripts, these ss-siRNAs effectively inhibited mutant RNAs in patient fibroblasts. Similarly designed ss-siRNAs were successfully applied to target expanded CUG repeats in the *TCF4* gene associated with Fuchs endothelial corneal dystrophy, significantly reducing RNA foci in cells with comparable efficacy to LNA-modified steric-blocking ASOs [168]. In this case, fully complementary ss-siRNAs demonstrated superior potency compared to mismatch-containing variants, highlighting the importance of optimal base pairing. Remarkably, in both therapeutic applications, the applied variant of ss-siRNAs constructs demonstrated inhibitory activity that matched or exceeded that of analogous double-stranded siRNAs targeting identical repeat regions [166,168].

Terminal region modifications significantly impact association with AGO and ss-siRNA activity in a target-dependent manner. At the 3′-end, substituting of traditional 2′-O-MOE-adenosines with 2′-OMe- or LNA-adenosines has been shown to enhance inhibitory efficiency for specific targets (Figure 5b(1),c) [167]. The 2′-OMe-modified 3′-diadenine variant inhibited asthma-associated *ADAM33* mRNA with efficiency comparable to ds-siRNA and 2.5-fold higher than other analogs in lung fibroblasts, while LNA-modified 3′-ends showed superior activity for progesterone receptor suppression in breast cancer cells. These findings indicate a probable sequence-dependent relationship between ss-siRNA design and activity for specific targets. Concurrently, optimization of the 5′-region by introducing 3′-deoxythymidine (3dT) at the first position significantly increased RISC affinity compared to traditional 2′-OMe-uracil modifications (Figure 5b(2),c) [169]. When combined with LNA-modified nucleotides at positions 3, 5, and 7 in the seed region, this design provided a 60-fold increase in β-catenin *CTNNB1* mRNA suppression in mouse liver cells compared to conventional ss-siRNA, achieving efficiency comparable to ds-siRNA. Lipid nanoparticle delivery of these 5′-optimized ss-siRNAs demonstrated significant target suppression in mouse liver in vivo, only slightly inferior to ds-siRNA [169].

Modified ss-siRNAs have demonstrated therapeutic potential against infectious diseases. A DNCA/CLD lipid-based delivery system was developed for targeting hepatitis B virus *X* gene mRNA, utilizing ss-siRNAs with alternating 2′-F/2′-OMe modifications, terminal phosphorothioate bonds, and a 5′-natural phosphate (Figure 5b(3),c) [170]. This design achieved 80% AGO2-mediated target suppression in infected cells, twice exceeding the efficacy of unmodified oligonucleotides, though tissue biodistribution studies revealed lower stability compared to ds-siRNAs. For cryptosporidiosis treatment, an innovative strategy was employed involving the direct introducing of ss-siRNAs targeting the nucleoside diphosphate kinase gene as part of pre-formed complexes with AGO2, since Cryptosporidium parasites lack endogenous RISC machinery [174]. This minimal RNA interference system effectively reduced parasite load in human intestinal epithelial cells in vitro and eliminated parasites in mouse models in vivo.

#### 4.2.2. Single-Stranded miRNA Mimics

A promising therapeutic approach utilizing short single-stranded RNAs as inhibitors involves developing ss-miRNA mimics that simulate mature miRNAs and suppress target gene expression through the endogenous RNAi pathway via interaction with AGO2 protein [163,171] (Figure 5d). While the predominant strategy for restoring miRNA expression that has been diminished or lost due to pathological processes has involved the application of duplex miRNA mimics, research focused on developing single-stranded mature miRNAs remains limited. Synthetic ss-miRNAs are constructed according to fundamental miRNA functioning principles and, unlike ss-siRNAs, these sequences are not fully complementary to target mRNAs [163,171] (Figure 5d,e).

Single-stranded mimics of tumor suppressor miRNAs miR-34a and let-7a with chemical modifications were developed to provide nuclease resistance while maintaining RNAi functionality. These ss-miRNA mimics incorporated 5′-phosphate, 2′-O-MOE-modified terminal nucleotides, and alternating 2′-F/2′-OMe ribose modifications with mixed phosphorothioate/phosphodiester bonds (Figure 5c,e(1)) [163]. Interestingly, the efficiency of ss- and ds-mimics varied significantly depending on the target. Both ss and ds miR-34a mimics effectively suppressed expression of *SIRT1*, *ARHGAP1*, *E2F5*, *CDK6*, *CDK4*, *MET*, *CCND1*, *E2F3*, *NOTCH2*, *AXL*, and *SNAI1* target genes in HeLa cells. *CCNE2* was uniquely suppressed by ss-miRNA mimics, while ds-mimics showed greater efficacy against *FOSL1*, *SIRT1*, and *CD44*. The let-7a ss-mimic demonstrated lower efficiency than its ds-counterpart for *FIGN*, *PLAGL2*, and *HMGA2* suppression. Activity of these mimics was dependent on both AGO2 expression levels and AGO2-miRNA binding efficiency. Successful therapeutic application was demonstrated for synthetic ss-miR-216b containing unlocked nucleic acid (UNA) modifications at positions 11 and 22 in pancreatic ductal adenocarcinoma (Figure 5c,e(2)) [171]. When delivered via TAT peptide-functionalized POPC liposomes in Panc-1 cells, this construct achieved 90% reduction in KRAS protein levels through an AGO2-dependent mechanism providing 50% suppression of colony formation.

#### 4.2.3. Small tRNA-Derived RNA Mimics

**Figure 6:**
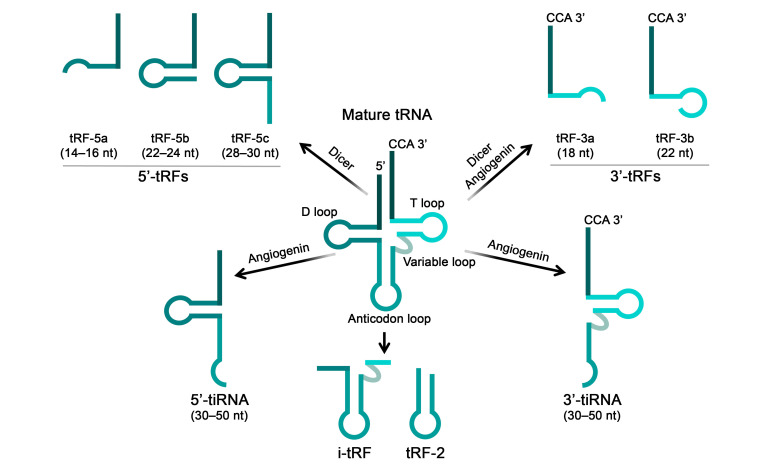
Biogenesis of transfer RNA-derived small RNAs (tsRNA). Schematic representation of tsRNA generation from mature tRNA. This process produces distinct tsRNA classes, including transfer RNA-derived fragments (5′-tRFs, 3′-tRFs), tRNA-derived stress-induced RNAs (5′-tiRNA, 3′-tiRNA) and internal fragments (i-tRF and tRF-2).

Proteins of the AGO subfamily can participate in gene regulation not only through interactions with miRNAs, siRNAs, and tyRNAs, but also with other small RNAs such as tsRNAs. In recent years, tsRNAs have attracted increasing attention due to the discovery of their important regulatory role in maintaining cellular homeostasis and their potential application in diagnostics and development of novel therapeutic agents [175,176,177,178]. tsRNAs represent a class of small non-coding RNAs generated through cleavage of various regions of mature tRNAs or their precursors by such ribonuclease as angiogenin, Dicer, RNase Z, RNase P and others (Figure 6). Depending on their length and mechanism of generation, tsRNAs are divided into two main groups: tRNA-derived stress-induced RNAs (tiRNAs) with lengths of 31–40 nucleotides, and tRNA-derived fragments (tRFs) with lengths of 14–30 nucleotides, which are further subdivided into several types based on the part of tRNA molecule from which they originate (Figure 6). tsRNAs are involved in the regulation of a broad spectrum of biological processes, including gene expression, translation, post-transcriptional modifications, and others (reviewed in [179]). According to the latest data tsRNAs can inhibit gene expression utilizing miRNA-like mechanism, wherein tsRNAs interact with AGO proteins, form the RISC, and subsequently create a complementary association between their seed region and the 3′-UTR of the particular mRNA molecule, thereby inhibiting its expression (Figure 5f) [24]. AGO proteins perform a protective function by shielding tsRNAs from degradation; notably, AGO2 has been shown to mediate the transport of associated tsRNAs from the extracellular environment into the intracellular space [180].

Aberrant tsRNA biogenesis and homeostasis, characterized by either elevated or reduced tsRNA levels, can be associated with the development of pathological conditions, including oncological [181,182], neurodegenerative [177], and cardiovascular diseases [178]. Factors influencing the levels of mature tsRNAs include the impaired activity of RNA polymerase III, which transcribes tRNA genes; post-transcriptional tRNA modifications; the level and activity of intracellular ribonucleases responsible for tRNA cleavage; the presence of ribonuclease inhibitors or modulating agents; translational activity; hormonal regulation; as well as various cellular stress conditions (for further details, see the review [183]). To restore pathologically reduced tsRNA levels and thereby suppress disease progression, researchers have employed synthetic analogs of these molecules—tsRNA mimics. The utilization of tsRNA mimics for delineating the functional significance and pathophysiological contributions of tsRNAs occurs simultaneously with the emergence of their therapeutic potential, establishing a promising area of research. As fragments of natural tRNAs, endogenous tsRNAs contain chemical modifications that support their integrity and stability [179]. However, for research purposes, unmodified tsRNAs analogs are predominantly employed, with their stability ensured by the delivery systems utilized.

Dysregulation of tsRNAs has been associated with the development of multiple malignant neoplasms. Specific alterations in the tsRNAs profile are characteristic of certain cancer cell types. For example, reduced levels of 5′-tRF and 3′-tRF derived from tRNA-Val were observed in breast [184], gastric [185,186], and colorectal cancer cells [187]; decreased levels of a 3′-tRF derived from tRNA-Glu were identified in cervical cancer cells [188], and lower abundance of 3′-tiRNA and 5′-tiRNA derived from tRNA-Asn and tRNA-Gly, respectively, was detected in gastric cancer cells [189,190]. Target mRNA suppression mediated by tsRNAs has been shown to involve AGO family proteins via an RNAi mechanism [186,187,188,190]. Transfection of tumor cells with synthetic mimics of tsRNA possessing tumor-suppressive properties leads to attenuation of malignant potential (Table 2). In particular, reduced cell proliferation, colony formation, migration, and invasion, as well as induction of apoptosis and cell cycle arrest, have been observed [184,185,186,187,188,189,190]. The magnitude of observed effects exhibited considerable variability depending on tumor type, specific cell line characteristics, molecular target, and applied mimic concentration. Collectively, the data indicate that, the most studies demonstrate that tsRNA mimics elicit modest antiproliferative (15–30%) and pro-apoptotic effects (increasing from 7–8% in control conditions to 10–16% following treatment), whereas their inhibitory impact on colony formation, migration, and invasion exhibits substantially more pronounced efficacy, reaching 50–75% inhibition (Table 2) [184,185,186,187,188,189,190]. The antitumor effect of tsRNA mimics was also confirmed in vivo using mouse xenografts models (Table 2) [185,186,187,190]. Upon treatment of gastric and colorectal tumor cells with tRFs (3′-tRF derived from tRNA-Val and 5′-tiRNA derived from tRNA-Gly), investigators achieved 50–70% tumor-suppressive efficiency utilizing mimics at a concentration of 0.05 µM, and near-complete tumor regression was observed following elevation of the concentration to 0.1 µM. It was revealed that the antitumor activity of tsRNA mimics is mediated via targeting of mRNAs involved in key signaling pathways that regulate tumor development. In particular, tsRNAs have been shown to modulate the THBS1/TGF-β1/Smad3 cascade associated with epithelial–mesenchymal transition [184], the PTEN/PI3K/AKT signaling pathway, controlling cell proliferation and differentiation [185], the canonical MAPK pathway (via *CACNA1d* mRNA), involved in proliferation control in certain carcinomas [186], and the Wnt/β-catenin signaling pathway (via direct targeting of the *FOXK1* transcript), associated with regulation of cell proliferation, differentiation, migration, and tissue homeostasis (Table 2) [187].

In the context of cardiovascular diseases, for instance, reduced levels of 3′-tRF derived from tRNA-Val and i-tRF from tRNA-Gln were reported in acute myocardial infarction and cardiac hypertrophy, respectively (Table 2) [191,192]. In a model of angiotensin II-induced hypertrophy, transfection of cardiomyocytes with a i-tRF mimic led to a decrease in the degree of cellular hypertrophy, and to a reduction in natriuretic peptide levels, specifically ANP by 40% and BNP by 70% [192]. Introduction of the 3′-tRF mimic into endothelial cells reduced *Tnfrsf10b* and *Bcl2l1* mRNA levels, potentially affecting signaling pathways related to lipid metabolism and atherosclerosis [191].

**Table 2:** Biological effects of tsRNA mimics in cellular and animal models.

tsRNA Mimic/Origin	Experimental Model and mRNA Targets (Signaling Pathway)	Cell Culture Effects *	In Vivo Effects *	Refs
5′-tRF (17 nt) **tRF-17-79MP9PP**/ tRNA-Val-CAG, tRNA-Val-AAC	Breast cancer: MCF-7, BT-549 Target: *THBS1* (THBS1/TGC/Smad3)	*THBS1* mRNA level ↓ 50–60% THBS1 protein level ↓ Proliferation ↓ 20–30% Colony formation ↓ 40–60% Invasion ↓ 65–70% Migration ↓ 65–75% Cell cycle: G1/S arrest	N.s.	[184]
5′-tRF (18 nt) **tRF-5026a**/ tRNA-Val-AAC	Gastric cancer: AGS, MGC-803, HGC-27, BGC-823, SGC-7901 Target: PTEN/PI3K/AKT	Protein levels: PI3K ↓ 25–60%, AKT ↓ 25–35%, PTEN ↑ 25–60% Proliferation ↓ 15–30% Colony formation ↓ 40–60% Migration ↓ 40–50% Cell cycle: G_0_/G_1_ arrest	SGC-823 and MGC-803 xenograft model: 0.05 µM mimic: tumor growth ↓ 50% 0.1 µM mimic: tumor growth inhibition in 5/6 mice	[185]
tRF (15 nt) **tRF-Val-CAC-016**/ tRNA-Val-CAC	Gastric cancer: NCI-N87, HGC-27 Target: *CACNA1d* (CACNA1d/MAPK)	*CACNA1d* mRNA level ↓ 30–45% Protein levels: CyclinD1, CyclinB, c-Myc ↓ Proliferation ↓ 65–80% Colony formation ↓ 70% Cell cycle: S arrest	NCI-N87 xenograft model: Tumor volume ↓ 50% Protein levels: CACNA1d, Ki-67 ↓	[186]
3′-tRF (17 nt) **tRF-3008A**/ tRNA-Val	Colorectal cancer: HCT116 Target: *FOXK1* (Wnt/β-catenin)	Proliferation ↓ 20% Invasion ↓ 40% Migration ↓ 50% Apoptosis ↑ 60%	In vitro treatment of HCT116 cells with subsequent implantation into mice: Tumor volume ↓ 50% Metastases ↓ Protein levels: Caspase-3, Ki-67, MMP9 ↓	[187]
3′-tRF (27 nt) **tRF-Glu49**/ tRNA-Glu-TTC, tRNA-Glu-CTC	Cervical cancer: HeLa Target: *FGL1*	*FGL1* mRNA level ↓ 60% Proliferation ↓ 25% Invasion ↓ 60% Migration ↓ 50%	N.s.	[188]
3′-tiRNA (41 nt) **tRF-41-YDLBRY73W0K5KKOVD**/ tRNA-Asn-GTT	Gastric cancer: HGC-27, AGS Target: *PAPSS2*	Proliferation ↓ 25% Migration ↓ 50–60% Cell cycle: G0/G1 arrest Apoptosis ↑ 40%	N.s.	[189]
5′-tiRNA (33 nt) **tRF-33-P4R8YP9LON4VDP**/ tRNA-Gly-GCC	Gastric cancer: HGC-27, AGS Target: *STAT3*	Proliferation ↓ 25% Colony formation ↓ 20% Invasion ↓ 30–40% Migration ↓ 30–35% Apoptosis ↑ 1.3–2.5-fold	In vitro treatment of HGC-27 cells with subsequent implantation into mice: 0.05 µM mimic: tumor growth ↓ 60–70% 0.1 µM mimic: tumor growth ↓ 90–97%	[190]
3′-tRF (17 nt) **tRF-60:76-Val-AAC-1-M5**/ tRNA-Val-AAC	Angiogenesis model: HUVEC Target: *Tnfrsf10b*, *Bcl2l1*	mRNA levels: *Tnfrsf10b* ↓ 10%*,* *Bcl2l1* ↓ 7%	N.s.	[191]
i-tRF (21 nt) **tRF-21-NB8PLML3E**/ tRNA-Gln-CTG	Cardiac hypertrophy: angiotensin II-stimulated H9c2 cardiomyocytes	Cardiomyocyte hypertrophy ↓ Natriuretic peptides levels: ANP ↓ 40%, BNP ↓ 75%	N.s.	[192]
3′-tRF (17 nt) **tRF3-Thr-AGT**/ tRNA-Thr-AGT	Cellular acute pancreatitis: AR42J rat pancreatic acinar cells treated with sodium taurocholate Target: *ZBP1* (ZBP1/NLRP3)	Proliferation ↑ 60% ^#^ Pro-inflammatory cytokines levels: IL-1β ↓ 55–65% and IL-18 ↓ 65% ^#^ Proteins levels: NLRP3, ASC, Gasdermin-D ↓ 40–50% ^#^, Caspase-1 ↓ ^#^	N.s.	[193]
3′-tRF (17 nt) **tRF-3003a**/ tRNA-Cys-GCA	Osteoarthritis: IL-1β-treated TC28/I2 chondrocytes Target: *JAK3* (JAK/STAT)	*JAK3* mRNA level ↓ 90% IL-6 protein level ↓ 65%	N.s.	[194]

* all values represent changes relative to the control mimic unless otherwise stated. # values represent changes relative to the sodium taurocholate-treated cells. “N.s.” indicates that corresponding studies were not performed. All experiments were conducted using commercial transfection reagents Lipofectamine or X-TremeGENE siRNA transfection reagent. Light green–oncological models; light orange–cardiovascular models; light blue–inflammation models. Arrows represent the direction of change: ↑ = increase; ↓ = decrease.

tsRNAs are also implicated in the regulation of inflammatory processes (Table 2). For example, reduced levels of 3′-tRFs derived from tRNA-Cys and tRNA-Thr were detected in chondrocytes in IL-1β-induced osteoarthritis and in pancreatic acinar cells in a model of acute pancreatitis [193,194]. Introduction of tRNA-Cys-derived 3′-tRF mimic into chondrocytes led to decreased *JAK3* mRNA expression [194]. Transfection with tRNA-Thr-derived 3′-tRF mimic restored cell viability, normalized inflammatory response, and induced pyroptosis-related protein expression via *ZBP1* mRNA degradation and inactivation of the pro-inflammatory ZBP1/NLRP3 signaling pathway [193].

## 5. PIWI Proteins as Key Mediators of RNA-Inhibitory Action of piRNAs

### 5.1. Structure, Mechanism of Action, and Primary Functions of PIWI Proteins

**Figure 7:**
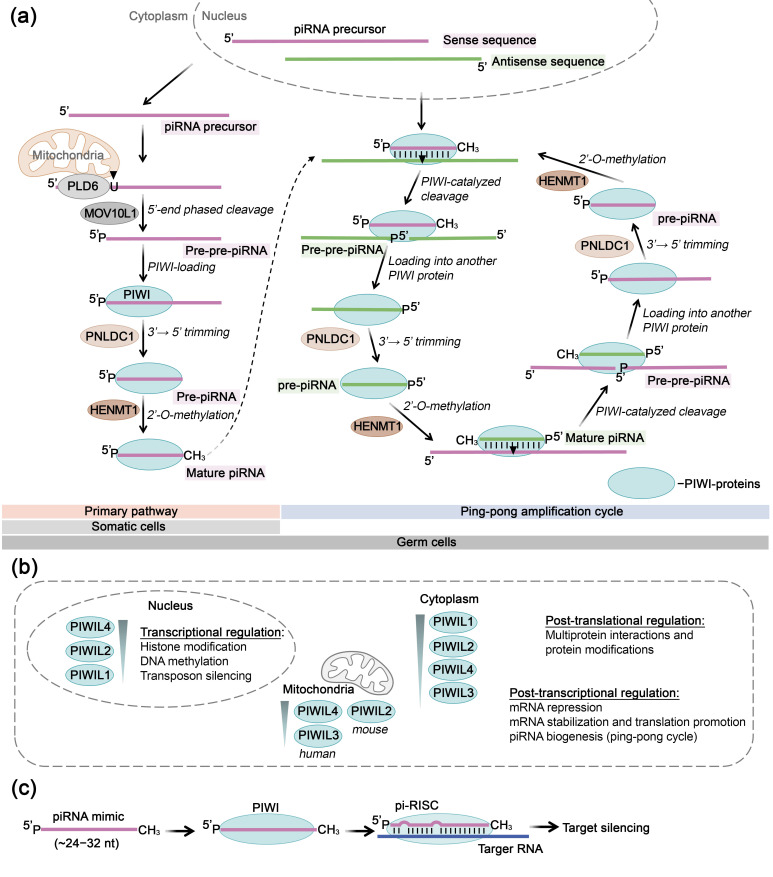
piRNA biogenesis and compartment-specific functions of PIWI family proteins in mammals. (**a**). piRNA biogenesis and processing pathway. piRNA production occurs through two main pathways: (1) primary processing begins with transcription of piRNA clusters followed by PLD6 endonuclease cleavage with MOV10L1 helicase assistance, generating 5′-monophosphorylated fragments that are loaded onto PIWI proteins. These fragments are then 3′-trimmed by PNLDC1 exonuclease and 2′-O-methylated by HENMT1, yielding mature primary piRNAs that mediate both transcriptional and post-transcriptional gene silencing; (2) secondary biogenesis occurs via the ping-pong amplification cycle, primarily active in germline cells and absent or reduced in most somatic tissues. In this pathway, PIWI proteins (PIWIL2 and PIWIL4) guided by piRNAs cleave target transcripts, generating new 5′-monophosphorylated piRNA precursors that are processed and loaded onto another PIWI protein for reciprocal targeting, establishing a self-amplifying piRNA production system and creating mature secondary piRNAs for functional gene regulation [195,196]. (**b**). Subcellular functional specialization of PIWI proteins. Compartmentalization represents predominant enrichment patterns based on functional specialization; however, spatial specification is highly generalized since PIWI localization is dynamic and context-dependent [25,26,196,197]. (**c**) Scheme of target RNA suppression by piRNA involving PIWI proteins. piRISC—piRNA-induced gene silencing complex; P–5′ monophosphate; CH_3_–2′-O-methyl group; U–uridine at 5′ end, preferred cleavage site for PLD6.

PIWI proteins belong to the Argonaute family and serve as key mediators of the function of piRNAs. piRNAs represent a class of small non-coding RNAs approximately 24–32 nucleotides in length that interact with PIWI proteins to form piRNA/PIWI complexes, playing crucial roles in regulating germline stem cell vitality, protecting the genome from mobile elements, controlling epigenetic regulation, and managing protein activity. piRNAs are generated from long single-stranded RNA precursors transcribed from specific genomic loci. The processing of piRNA precursors involves a complex multi-stage process that occurs through two main pathways: primary processing and ping-pong amplification, requiring various enzymes and results in the formation of mature piRNA molecules, which contain uracil residue at the 5′-end and 2′-OMe modification at the 3′-end (Figure 7a). The biogenesis of piRNAs is extensively reviewed in [195,196]. Unlike AGO proteins, which are ubiquitously expressed, PIWI proteins are predominantly expressed in germline stem cells [22,23]. Nevertheless, experimental evidences indicating the functional significance of piRNAs in somatic cells continue to accumulate steadily [18,19,20,21].

The structure and functions of PIWI subfamily proteins are highly conserved across different organisms. Four homologs have been identified in human tissues: PIWIL1 (HIWI), PIWIL2 (HILI), PIWIL3 (HIWI3), and PIWIL4 (HIWI2) [198]. PIWI proteins consist of a variable N-terminal domain, central PAZ and MID domains, and a C-terminal PIWI domain possessing endonuclease activity (Figure 4). The PAZ domain recognizes the 2′-OMe 3′-end of piRNAs, while the MID domain binds to the phosphorylated 5′-end of the molecule [199].

The functional significance of piRNAs manifests at transcriptional, post-transcriptional, and post-translational levels (Figure 7b) [25,26,196,200]. At the transcriptional level, the piRNA/PIWI complex executes gene suppression (transcriptional gene silencing) through DNA methylation and histone modification. Post-transcriptional suppression of RNA activity occurs via interaction of the piRNA/PIWI complex with target lncRNAs or mRNAs, forming a piRNA-induced gene silencing complex (piRISC) that degrades RNA through a miRNA-like mechanism (Figure 7c). To achieve target cleavage, PIWI proteins, unlike AGO proteins, require extended but more flexible piRNA base pairing with the target RNA. Experiments with mouse PIWI proteins demonstrated that PIWI-mediated slicing does not depend on strict complementarity in the seed region (2–8 nt of piRNA), and even mismatches at the cleavage site (target positions 10 and 11) are tolerated. However, efficient cleavage generally requires at least 15 contiguous base pairs between the piRNA and the target [201]. This mismatch tolerance provides the piRNA pathway with adaptability, enabling efficient recognition and silencing of rapidly evolving transposons. At the post-translational level, the piRNA/PIWI complex controls the functional activity of transcription factors through phosphorylation and ubiquitination. While PIWIL1, PIWIL2, and PIWIL4 are widely expressed across various developmental stages and tissues in both germline and somatic cells, PIWIL3 shows more restricted expression and is specifically found in maturing human oocytes during adult oogenesis [202]. Functionally, PIWIL1 and PIWIL2 predominantly participate in transcript degradation in the cytoplasm at the post-transcriptional level, PIWIL3 operates exclusively in oocytes for cytoplasmic transcript regulation, whereas PIWIL4 participates in transcriptional gene silencing in the nucleus [26].

The growing interest in piRNAs stems from their involvement in key biological processes, including the regulation of cell proliferation, metabolism, cell cycle progression, activation of invasion and metastasis, induction of apoptosis, and immune modulation. Consequently, dysregulation of piRNA expression is associated with the development of pathological conditions, highlighting their potential as diagnostic biomarkers and therapeutic targets.

### 5.2. PIWI-Mediated RNA Inhibition Using Synthetic piRNAs

Similarly to previously discussed small RNAs such as miRNAs and tsRNAs, piRNAs are involved in post-transcriptional gene silencing and their dysregulation, manifested as either reduced or elevated level, has been associated with the development of various pathological conditions including cancer, cardiovascular disorders, disease of the nervous and reproductive systems and viral infections [21]. The use of synthetic piRNA mimics to restore the function of downregulated piRNAs, in combination with PIWI proteins, represents a promising therapeutic approach (Figure 7c).

For example, a recent study demonstrated that in pancreatic tumor cells reduced level of piR-017061 has been observed and transfection with a 2′-OMe-modified mimic suppressed cell growth by ~30% and colony formation by 50% in vitro and inhibited tumor growth by up to 90% in xenografted mice models accompanied by significant induction of apoptosis. piR-017061 interacts with PIWIL1 to mediate the degradation of target *EFNA5* mRNA [203]. Similarly, in clear-cell renal carcinoma cells, downregulation of piR-57125 was reported. Cell transfection with synthetic mimic led to a decrease in migration and invasion by 40–60%, without affecting proliferation. Mechanistically, piR-57125 binds the 3′-UTR of *CCL3* mRNA and suppresses it via a PIWIL4-dependent RISC pathway. *CCL3* inhibition disrupts the AKT/ERK signaling cascade, involved in metastatic progression [204].

The application of piRNA mimics has also been recently investigated in the context of antiviral therapy. Transfection of the human lung fibroblast cells with piRNA-hsa-28,382 mimic followed by HSV-1 infection reduced viral titer by 15% [205]. Bioinformatic analysis suggested that piRNA-hsa-28,382 may target viral genes *RL2*, *RS1*, and *UL54*.

The potential of PIWI proteins also was explored using cells transfected with vector expressing therapeutic piRNAs. piR-19166, downregulated in prostate carcinoma cells, inhibited cell migration by up to 40% in vitro and reduced metastasis by 75% in a mouse model through suppression of *CTTN* mRNA, a key component of CTTN/MMPs signaling pathway responsible for metastasis [206]. Similarly, piR-18 expression in colorectal cancer cells reduced proliferation by ~20% and invasion by ~40% in vitro, and tumor growth by ~40% in vivo [207].

## 6. Conclusionss

Endogenous ribonucleases, including RNase H1, RNase P, and proteins of the AGO family, play a fundamental role in mediating antisense-induced inactivation of target RNA molecules. The effective implementation of these technologies relies on a comprehensive understanding of the functional mechanisms of the employed enzymes, which subsequently facilitates the development of highly efficient strategies of RNA activity modulation for therapeutic applications.

It is evident that numerous molecular and structural parameters critically modulate the efficiency of antisense inhibition, encompassing a complex interplay of factors: the selection of RNA target sequences, structural accessibility and intracellular localization of RNA targets, enzyme-specific compartmental distribution (for RNase H1, RNase P, and AGO protein family), molecular architecture and pattern of chemical modification of therapeutic nucleic acids, and the molecular mechanisms of target-specific delivery systems.

Antisense oligonucleotides represent the most versatile molecular tool, strategically designed to target virtually any intracellular RNA sequence across diverse molecular classes (mRNA, pre-miRNA, miRNA, lncRNAs, piRNAs, circRNAs, and others), considering their structural accessibility. The ubiquitous localization of RNase H1 across nuclear, cytoplasmic, and mitochondrial compartments, coupled with the extensive RNA target diversity, establishes this technology as a highly adaptable and potent molecular strategy. The approach employing EGS facilitates co-localization of therapeutic nucleic acids with RNase P predominantly within the nuclear compartment, primarily targeting viral and bacterial transcripts.

Single-stranded small RNA mimics (miRNA, tsRNA, and piRNA mimics), due to their homology to endogenous molecules, are mediating post-transcriptional gene silencing in cooperation with proteins of the AGO family, predominantly within the cytoplasm. Among PIWI proteins, PIWIL1 and PIWIL4 are most prominently involved in mediating RNAi employing synthetic piRNAs. Notably, the dynamic translocation capabilities of AGO proteins between nuclear and cytoplasmic compartments provide a strategic mechanism for expanding the potential RNA target repertoire [208]. While miRNAs primarily repress gene expression through translational inhibition and RNA degradation, other small RNAs, such as piRNAs and tsRNAs, may exert broader regulatory functions, including chromatin remodeling and transcriptional silencing, operating both in the cytoplasm and nucleus. Although AGO subfamily proteins, particularly AGO2, have been extensively characterized at the structural and functional level, studies on human PIWI proteins remain limited. To date, only isolated domains of PIWI proteins have been structurally resolved, primarily for the purpose of elucidating their specific molecular functions, whereas full-length structures are still lacking. Further characterization of PIWI proteins, particularly their compartment-specific activities and mechanistic role, promises to reveal their additional functional load and therapeutic potential.

To achieve optimal therapeutic efficiency, delivery systems must be engineered to ensure precise co-localization of therapeutic nucleic acids in proximity to target RNA and associated enzymatic systems. Despite decades of intensive research demonstrating substantial progress, the challenge of targeted drug delivery has not been fully resolved, representing a critical frontier in molecular therapy so far.

The overall structural architecture and chemical composition of therapeutic oligonucleotides fundamentally govern their biological performance. The therapeutic potential of antisense oligonucleotides and single-stranded RNAs has been comprehensively investigated. Extensive research has focused on structural optimization, developing and implementing diverse chemical modifications and their compositions to enhance molecular biocompatibility and functional properties. In order to promote RNase H1 recruitment capabilities, chemical modifications must preserve the geometric integrity of the heteroduplex formation with target RNA. While researchers have developed a substantial arsenal of chemical modifications, the search for optimal structural variants continues in pursuit of improved nuclease resistance, reduced immunogenicity, and minimal toxicity while maintaining high therapeutic efficiency.

The exploration of therapeutic potential for tsRNA and piRNA mimics represents an emerging research area. Due to the limited understanding of how chemical modifications affect the activity of these molecules, researchers predominantly investigate therapeutic nucleic acids in their native compositional state, without additional chemical alterations. However, it can be expected that in the near future synthetic tsRNA and piRNA analogs with improved chemical and biological properties will be developed.

It should be noted that the study of the therapeutic potential of non-coding RNAs, particularly tsRNAs and piRNAs in association with AGO and PIWI subfamily proteins have been predominantly investigated in the context of cancer. This trend likely reflects the widespread availability and experimental tractability of tumor models, which offer convenient systems for evaluating molecular function and therapeutic potential, especially at early stages of research. However, the spectrum of pathologies that can be addressed by these emerging therapeutic strategies is expected to continuously expand. As our understanding of the tissue-specific roles of tsRNAs and piRNAs deepens, the therapeutic potential for these molecules is expected to broaden substantially beyond oncology applications.

**Figure 8:**
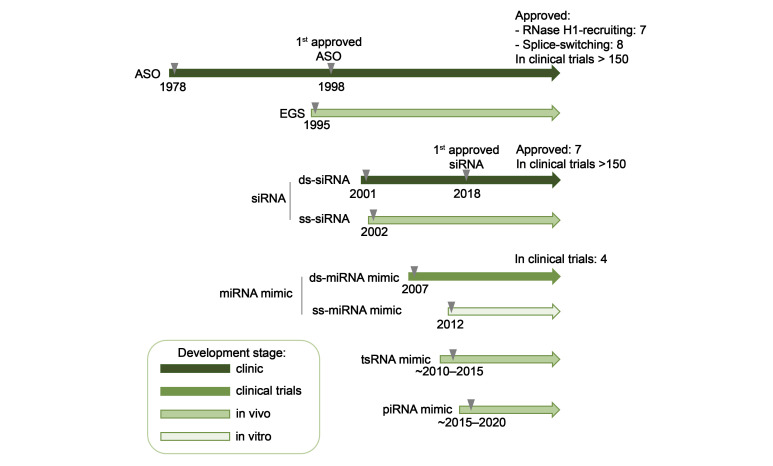
Clinical translation progress of ribonuclease-recruiting oligonucleotide therapies. The chronological scheme shows years of initial application and current development stages illustrated by coloring from preclinical research (light) to clinical application (dark). ASO—antisense oligonucleotides; EGS—external guide sequences; siRNA—small interfering RNA; ds and ss—double-stranded and single-stranded; tsRNA—tRNA-derived small RNA; piRNA—PIWI-interacting RNA.

The utilization of endogenous enzymatic systems for therapeutic development has yielded successful outcomes, with antisense oligonucleotides representing the most advanced technology. As illustrated in Figure 8, the development of the first approved nucleic acid-based therapeutics required 20 years of research. Currently, 15 antisense oligonucleotide drugs have reached clinical application: 7 RNase H1-recruiting gapmer compounds and 8 splice-switching oligonucleotides, with over 150 additional drugs in clinical trials. RNA interference using double-stranded siRNAs has resulted in 7 approved drugs since 2001, with over 150 more in trials (Figure 8). Single-stranded siRNAs are currently at the preclinical research stage but offer significant advantages including reduced off-target effects and simplified delivery. Similarly, double-stranded miRNA mimics have progressed to clinical trials (Figure 8), while single-stranded structures are currently at the in vitro stage but may demonstrate enhanced specificity and reduced immunogenicity. The tsRNA/AGO technology, originating in 2010–2015, has generated successful preclinical results with promising prospects for clinical translation. The piRNA/PIWI partnership represents an even more emerging field, with therapeutic applications of this partnership only beginning to be explored. Current research focuses on both piRNA suppression and piRNA mimics applications. The EGS/RNase P approach, despite extensive research history, lags behind other technologies (Figure 8), likely due to nuclear enzyme localization creating delivery and functionality challenges compared to cytoplasm-active technologies. The pioneering success of antisense oligonucleotides, with extensive research that has generated valuable insights into efficacy optimization, immunogenicity management, and toxicity assessment, can accelerate the development of other nucleic acid technologies. This knowledge base allows researchers to develop efficient drugs based on established principles while addressing the specific requirements of each emerging therapeutic approach.

A promising complementary technology to endogenous enzymatic systems involves the development of hybrid approaches representing sequence-specific artificial ribonucleases (aRNases). These compounds integrate an oligonucleotide recognition domain ensuring target RNA identification with a catalytic domain of natural origin, thereby enabling autonomous RNA targeting and degradation [209,210,211,212,213,214,215]. The additional recruitment of RNase H1 substantially amplifies the targeted RNA destruction [210,216], illustrating how natural ribonuclease principles can be enhanced to create more effective therapeutic approaches.

In conclusion, the integration of endogenous ribonucleases with therapeutic nucleic acids represents a unique, high-specific therapeutic approach that maximally harnesses the endogenous enzymatic potential of cellular systems. This strategy is characterized by high biological complementarity, as it exploits intrinsic cellular metabolic mechanisms, thereby ensuring minimization of adverse effects, enhanced selectivity of action, and the creation of a maximally autonomous, physiologically integrated therapeutic modality.

## Data Availability

No new data were created in this study.

## Abbreviations

The following abbreviations are used in this manuscript:

2′-F-tc-ANA	2′-β-fluoro-tricyclo-arabinonucleic acid
2′-F	2′-fluororibose
2′-F-ANA	2′-deoxy-2′-fluoro-β-d-arabinonucleic acid
2′-OMe	2′-O-methyl
2′-O-MOE	2′-O-methoxyethyl
3′-UTR	3′-untranslated region
3dT	3′-deoxythymidine
4′-(*S*)-2-APoT and 4′-(*R*)-2-APoT	4′-C-2-aminopropoxy-modified thymidine in *S*- and *R*-configuration
5′-(E)-VP	5′-(E)-vinylphosphonate
5′-HP	5′-hydroxyphosphonate
5′-MEP	5′-O-methylenephosphonate
6′-diF-bc4,3-DNA	6′-difluorinated[4.3.0]bicyclo-nucleotides
6′-F-bc4,3-DNA	6′-fluoro[4.3.0]bicyclo-nucleotides
AcPO	Phosphonoacetate
AcPS	Thiophosphonoacetate
ADAM33	Adam metallopeptidase domain 33
AEoT	4′-C-aminoethoxy thymidine
ALS	Amyotrophic lateral sclerosis
ANA	Arabinonucleic acid
ANP	Atrial natriuretic peptide
ANXA2	Annexin A2
ApoB-100	Apolipoprotein B-100
Apo-CIII	Apolipoprotein CIII
araU^P^ and araC^P^	C5-propynyl arabinouridine and arabinocytidine
ASO	Antisense oligonucleotide
Bcl2l1	Bcl-2-like protein 1
BNP	B-type natriuretic peptide
CACNA1d	Calcium voltage-gated channel subunit alpha1 D
CAT	Catalytic domain
CCL3	CC motif chemokine ligand 3
CCR4-NOT	Carbon catabolite repression 4—negative on TATA-less
CCR5	CC-chemokine receptor 5
CD	Connecting domain
CeNA	Cyclohexenyl nucleic acid
cEt	Constrained ethyl nucleic acids
circRNA	Circular RNA
CTNNB1	Catenin beta 1
CTTN	Cortactin
DDX21	DExD-box helicase 21
DNCA/CLD	Neutral cytidine lipid/cationic lipid
EFNA5	Ephrin A5
EGS	External guide sequences
eIF4F	Eukaryotic initiation factor 4F
FGL1	Fibrinogen-like protein 1
FOXK1	Forkhead box protein K1
ftsZ	Filamenting temperature-sensitive mutant Z
gyrA	DNA gyrase subunit A
HBD	Hybrid-binding domain
HBV	Hepatitis B virus
HCMV	Human cytomegalovirus
HENMT1	HEN Methyltransferase 1
HIV	Human immunodeficiency virus
hnRNP	Heterogeneous nuclear ribonucleoproteins
hspA8	Heat shock protein family a (hsp70) member 8
HSV-1	Herpes simplex virus type 1
HSV-2	Herpes simplex virus type 2
HTT	Huntingtin
ICP8	Infected cell protein 8
IRES	Internal ribosome entry site
JAK3	Janus kinase 3
KRAS	Kirsten rat sarcoma virus
LDL	Low-density lipoprotein
LNA	Locked nucleic acids
lncRNA	Long non-coding RNA
MALAT1	Metastasis associated lung adenocarcinoma transcript 1
MCP	Major capsid protein
MID	Middle domain
miRNA	MicroRNA
MOV10L1	Mov10-like RNA Helicase 1
MTS	Mitochondrial targeting sequence
n.s.	Not studied
NAT10	N-acetyltransferase 10
NPC1	Niemann-pick C1
NPM1	Nucleophosmin 1
P54nrb protein	54-kda nuclear RNA-binding protein
PAPSS2	3′-phosphoadenosine 5′-phosphosulfate synthetase 2
PAZ	PIWI-Argonaute-Zwille
PC4	Positive cofactor 4
PF	Phosphonoformate
piRISC	PiRNA-induced gene silencing complex
piRNA	PIWI (P element induced wimpy testis)-interacting RNA
PIWI	P element induced wimpy testis
PLD6	Phospholipase D Family Member 6
PNLDC1	PARN Like Ribonuclease Domain Containing Exonuclease 1
POPC	Palmityl-oleyl-phosphatidylcholine
pre-tRNA	Precursor transfer RNA
PS	Phosphorothioate
PTEN	Phosphatase and tensin homolog
PURPL	P53 upregulated regulator of p53 levels
PVT1	Plasmacytoma variant translocation 1
RISC	RNA-induced silencing complex
RNAi	RNA interference
SARS-CoV-2	Severe acute respiratory syndrome coronavirus 2
siRNA	Small interfering RNA
SOD1	Superoxide dismutase 1
SPS	Phosphorodithioate
ss-siRNA	Single-stranded siRNA
TAT	Transactivator of transcription
TCF4	Transcription factor 4
TCP1	T-Complex 1 protein
THBS1	Thrombospondin 1
tiRNA	tRNA-derived stress-induced RNA
TNA	Therapeutic nucleic acid
Tnfrsf10b	Tumor necrosis factor receptor superfamily member 10B
tRF	Transfer RNA-derived fragment
tRNA	Transfer RNA
tsRNA	Transfer RNA-derived small RNA
TTR	Transthyretin
tyRNA	Tiny RNA
UNA	Unlocked nucleic acid
VARS	Valyl-tRNA synthetase
ZBP1	Z-DNA binding protein 1
μ	Mesyl-(methanesulfonyl)-phosphoramidate
